# Constraints, synergies and avenues for scaling up breastfeeding, antibiotics for pneumonia and IMCI interventions in the Cusco region, Peru

**DOI:** 10.12688/f1000research.1-60.v1

**Published:** 2012-12-05

**Authors:** Giselle Sarganas, Robert Scherpbier, Christian A Gericke

**Affiliations:** 1 Berlin School of Public Health, Charite University Medicine, Berlin, 10117, Germany; 2 Health, Nutrition, Water, Environment & Sanitation, UNICEF, Beijing, 100600, China; 3 Peninsula CLAHRC, National Institute for Health Research, Peninsula Medical School, Plymouth University, Plymouth, PL4 8AA, UK; 4 The Wesley Research Institute, Brisbane, QLD 4066, Australia

## Abstract

**Objective: **The purpose of this qualitative case study was to assess the feasibility of scaling up exclusive breastfeeding for 6 months, antibiotics for pneumonia and integrated management of childhood illness (IMCI) child interventions in three districts of the Cusco region, Peru.

**Methods:** During field visits, constraints, synergies and solutions to the implementation of the selected interventions were collected through observational recording and interviews of mothers, health workers, and health managers/decision makers. Results are presented for each intervention according to the health system level where they occurred: mother/community, health worker, health centre, and political/managerial levels.

**Findings: **This case study demonstrates that it is feasible to scale up exclusive breastfeeding, antibiotics for pneumonia and IMCI interventions in poverty-stricken rural areas of a low-income country. Factors that helped and hindered the implementation were identified for each intervention.

**Conclusions: **The need for a coherent multi-sector approach that includes regulation, implementation and monitoring of health policies and education of all involved stakeholders was apparent. This study also demonstrates that global health interventions need to undergo local adaptation. Identifying local constraints and facilitating factors in a systematic way as proposed in this study is a useful step to increase their effectiveness and reach at the local level and to identify areas for improvement in the original intervention policies.

## Introduction

The global mortality rate in children younger than 5 years fell by 28%, from an estimated 90 deaths per 1000 live births in 1990, to 65 deaths per 1000 live births in 2008 ^1^. Still it was estimated that 7.2 million children under the age of five died in 2011 mostly because of treatable and preventable causes ^2^. Continued success towards achieving the Millennium Development Goal to reduce child mortality by two-thirds of the 1990 rate depends on renewed efforts to prevent and control pneumonia, diarrhoea, malaria and malnutrition ^1, 3^. UNICEF, the World Health Organization (WHO), and their technical partners, have developed the Integrated Management of Childhood Illness (IMCI) strategy, consisting of three components: improving the case management skills of health workers; improving the health system support needed for effective management of childhood illness; and promoting key family and community practices through the education of caretakers and other members of the community ^4^.

The Bellagio Child Survival Study Group and the Lancet Neonatal Survival Team have identified and evaluated 31 newborn and child preventive and treatment interventions ^5, 6^. Breastfeeding intervention emerged as the most important single intervention in terms of prevention, showing that if it is implemented with 90% coverage, it could reduce 13% of all under-five deaths. According to the Countdown Report (2010), the median coverage of exclusive breastfeeding in the 68 Countdown countries was only 34% ^1^. Antibiotics for pneumonia was identified as a treatment intervention, that if implemented with 99% coverage could reduce under-five mortality by 6% ^5, 6^. According to the Countdown Report (2010) the median coverage of antibiotics for pneumonia in the 68 Countdown countries was 27% ^1^. Although substantial progress has been made in reducing mortality and improving coverage, two major challenges remain: how to improve the quality of health interventions, and how to reach the most disadvantaged children ^7, 8^.

There is limited evidence on the process of implementing effective child health interventions, their cultural appropriateness, cost-effectiveness, and effects on health inequalities, all of which are important considerations for policy-making ^9^. It is not only a matter of how many children are receiving effective interventions; the quality with which these are delivered is also critical ^10^. To encourage the full implementation of health interventions, international recommendations need to be adapted locally, taking into account the political environment and the socio-cultural context. For that to happen, there is a need to evaluate how interventions are implemented, and which factors help and hinder their success. Child health interventions may vary substantially in the degree of effort to implement them, as implementation barriers may differ for different interventions. Every intervention requires for its implementation and sustainability both financial and non-financial resources that vary in terms of quality and quantity, known as intervention complexity ^11^.

The purpose of this study was to assess the feasibility to scale up exclusive breastfeeding for 6 months, antibiotics for pneumonia and IMCI child interventions in selected communities of the Cusco region, Peru.

In pursuing this purpose, the constraints, synergies and possible solutions to the implementation of these child interventions have been systematically assessed according to the conceptual framework developed by Gericke *et al.* ^12^.

## Methods

This is a qualitative exploratory case study which involves an empirical investigation of a contemporary phenomenon within its real life context using multiple sources of evidence in order to reach new insights and to assess phenomena in a new light ^13^. The purpose was to find out implementation characteristics of exclusive breastfeeding for 6 months, antibiotics for pneumonia and IMCI child interventions in order to scale them up. This field study was carried out in August 2007 in the Paucartambo, Canas and Calca districts of the Cusco region of Peru. These districts were selected as they have high under-five mortality rate in comparison with other districts in the region, are geographically representative (the regions are located in the North, South and Canchis Espinar areas, respectively) and should have an IMCI program based on their high under 5 mortality rates. One first-level facility per district (Huancarani, Yanaoca and Calca, respectively) was selected according to regional and local health manager consultations. The main characteristics of the selected districts and their health facilities are presented in Table 1.

**Table 1.** Health characteristics of selected districts and health facilities in three districts in the Cusco region, Peru.

Districts	Paucartambo	Canas	Calca
**Infant Mortality Rate** ^14^	39.8/1000	38.7/1000	28.5/1000
**Poverty Absolute Index** ^15^	Very poor	Very poor	Average
**Human Development Index** ^16^	0.43	0.45	0.50
**Under-5 Mortality causes** ^14^	-Perinatal pathologies -Traumatisms -Intoxications	-Perinatal pathologies -ARI	-Perinatal pathologies -ARI
**Health facilities**	**Huancarani**	**Yanaoca**	**Calca**
**Opening hours**	From 8 to 20 h. Emergencies: 24 h.	From 8 to 20 h. Emergencies: 24 h.	From 8 to 20 h. Emergencies: 24 h.
**Basic drugs, vaccines and equipment**	yes	yes	yes
**Number of health professionals**	8	5	12
**Number of communities under responsibility**	20	24	31
**Number of health posts in the surroundings**	4	10	6
**Local committee for shared resources administration** **within the community (CLAS)**	yes	yes	no
**Most frequent causes of under-5 morbidity**	-ARI -Undernutrition	-ARI -Undernutrition	-ARI -Undernutrition
**Most frequent causes of under-5 mortality**	-Perinatal asphyxia -Pneumonia	-Milk aspiration -Sepsis -Malformations	-Perinatal asphyxia -Sepsis -ARI

ARI: acute respiratory infections.

During the visits, the constraints, synergies, and solutions to the implementation of the selected child interventions were collected through observational recording and interviews with mothers, health workers, and health managers/decision makers.

A check list guide was created based on a previous systematic literature review in order to assist with the observational recording of health centres and their consultation characteristics, including a structured inventory of essential IMCI treatments, vaccines, and equipment. The child’s consultation was observed and recorded prior to the mother’s interview.

Standardized face to face in-depth interviews were performed with 11 mothers, nine health workers (three medical doctors, four nurses and two community health workers), and five health managers and decision makers. To standardize the interviews, an open questionnaire-guide tool in Spanish (see Interview and check list file) was created for each intervention based on the previous systematic literature review, and on the conceptual framework with its four dimensions: a) characteristics of the basic intervention, (basic product design, supplies and equipment) b) characteristics of delivery: facilities, human resources, communication and transport; c) requirements on government capacity: regulation/legislation, management systems and collaborative action; and d) usage characteristics (ease of usage, pre-existing demand and black market risk). Mothers were chosen on a convenience basis i.e. as long as they came with their children for the consultation. The child’s case management was observed prior to an interview with the mother. A local research assistant was appointed by the local collaborator to help with translation for mothers speaking the Quechua language. All interviews were audio-taped and transcribed. Full anonymity to all interviewees was guaranteed. Verbal consent was obtained from all interviewees.

Interview guide and checklist (Spanish) The interview guide and checklist used to record constraints, synergies and solutions to scaling up exclusive breastfeeding, antibiotics for pneumonia and intergrated management of childhood illness (IMCI) interventions in the Cusco region, Peru.

The constraints, synergies, and solutions from the observational recordings and interviews of the three key informant groups were labelled and classified according to the level where they were found to be present and through the application of the selected conceptual framework. The constraints and synergies were then counted to calculate their frequency (for each intervention the number of constraints or synergies adds up to 100%). Triangulation of information was used as a tool for testing one source of information against the others, considering that its by-products were as useful as its primary purpose in validating information.

## Results

Results are presented for each intervention according to the level they were found to be present: mother/community, health worker and political/managerial levels. For the IMCI intervention, the same levels were identified and a health centre level was added. The frequency of detected constraints and synergies guided the presentation’s order.

## Exclusive breastfeeding for 6 months intervention

### Mother/community level

Most barriers (55%) to exclusive breastfeeding (EBF) for 6 months correspond to the mother/community level ( Table 2). Even though the majority of the mothers are aware that health workers recommend EBF for 6 months, find it easy to breastfeed, and have support from their partners, they still practice mixed feeding in the first 6 months of the infant’s life. A large proportion of the interviewed mothers had also introduced mate (a traditional sort of tea in the region), either alone, or with other foods before the 6th month of life. Some of their reasons for giving mate included treating their baby’s abdominal spasms, the baby’s preferences, and not having enough maternal milk. The reason for not having enough breast milk also leads to the early introduction of cow’s milk; one mother responded, "I don’t have enough milk, because it is my 7th child that is why I help myself with cow milk". Mothers also justify this practice by arguing that cow’s milk is better than their own milk.

**Table 2.** Classification of identified exclusive breastfeeding constraints and synergies according to their target level and label together with the percentage of respondents that agreed with each label.

Target level	Constraint’s label	%	Synergy’s label	%
**Mother/community**	Mother’s knowledge, beliefs, attitudes and practices	46	Mothers knowledge, beliefs, attitudes and practices	28
	Mother’s low economical resources, poverty	6		
	Mother’s low education	3		
**Health worker**	Field activities, community component	10	Field activities, community component	20
	Health workers performance	8	Health workers performance	37
	Lack of human resources specific training	5	Health workers training	3
	Lack of measurement of mothers’ practices	3		
	Time constraints for counselling	2		
**Political/managerial**	Lack of supportive work regulations	18	Supportive policies and collaborative actions	11
			BF promotion while delivery of food products	1
			Health workers surveys	1

EBF practice is also affected by a mother’s educational level and nutritional status; some examples of health worker responses were "mothers tend to forget what we, health workers, have said", and "it’s not difficult to breastfeed, but if I don’t eat I don’t have milk". Not giving colostrum to the baby or delaying breastfeeding, particularly in home deliveries, have been reported by health workers and managers. Even though they acknowledged that these practices have been partially overcome by the institutionalisation of deliveries, they are still present as highlighted by responses such as, "the mother is considered more important than the baby and that is why in the first 6–7 hours after delivery they don’t practice breastfeeding", and "mothers think that the newborn does not need to be breastfed right away".

The solutions proposed by health professionals and managers to overcome these obstacles include mother and community education, addressing knowledge, beliefs, attitudes and practices, and increasing promotion of EBF for 6 months by mainly designating specific local health workers, but also including all stakeholders, to increase awareness. For that to occur, informative printed media and audio-visual kits in waiting rooms of health establishments, or community education programs such as socio-drama (market theatre) is needed.

### Political/managerial level

18% of reported obstacles correspond to the political/managerial level under the label ‘lack of supportive work regulations’. This constraint is found throughout working mothers, including health professionals. Examples of responses provided in this respect include "we, as health staff, recommend to practice EBF for 6 months, but we don’t practice it because we can’t do that due to our own working conditions", and "even though there is a law that states the allowance of one hour per day for working mothers to breastfeed, nobody respects that".

The majority of mothers in this region work on farms and have the same workload as men. Health workers reported a low frequency of breastfeeding among these mothers; an example of one such response was that "mothers think the more the baby sleeps the better it is, so they can work more". Mothers have the socio-economic pressure to perform at work and the feeling that they should not waste their time. While some of them carry their babies on their back, others leave them at the farm or even at home and breastfeed when they return, as encapsulated by one response "I take my baby to the farm and breastfeed him while resting or when he cries".

According to the health centres’ observations, breastfeeding is promoted mainly by nurses who are in charge of monitoring the healthy child, while doctors focus more on the illness of the child. Supportive policies are trying to establish a specific place at health centres called ‘Lactarios’ where multidisciplinary teams made up of doctors, nurses, obstetricians and social assistants could educate and correct breastfeeding techniques when necessary. Proposed solutions by health managers include strengthening the implementation and monitoring of health policies, including the implementation of ‘Lactarios’, and the monitoring the compliance of health policies and regulations. Health managers consider themselves, as well as the Ministry of Labour and the Ministry of Women, as responsible for the implementation of these actions.

Other synergies in terms of collaborative actions such as the annual celebration of Breastfeeding Week in August, include support from NGOs who organize breastfeeding community lectures, and the Ministry of Health which supports and prioritises breastfeeding within the model of integral child care.

### Health worker level

Most detected synergies (60%) to EBF correspond to the health worker level ( Table 2). Health professionals reported that speaking the Quechua language is a facilitating factor when promoting breastfeeding while others believe that it should be a requisite for working in this area. Another facilitating factor to support communication and counselling to mothers is the role of community health workers (CHWs). According to health professionals, CHWs help address community beliefs and practices, stating that "thanks to the CHWs we can have more information about local practices", and "it is important to train CHWs for promoting an early breastfeeding practice".

Health worker actions for EBF intervention include promotion and education at health centres and in the community. Of the interviewed mothers, 50% had not received home visits, postnatal breastfeeding counselling, or had participated in community talks/lectures about breastfeeding. Some health workers acknowledged this fact, saying that "some home visits are performed, but not all".

Health workers and managers reported difficulties in knowing about mother’s practices, claiming that "even though mothers say that they practice EBF, we cannot prove whether it is done exclusively and with an adequate frequency", and "mothers say that they practice EBF, but we really don’t know if they give to the babies also mate or water".

During field observations, breastfeeding techniques were not regularly checked, but 70% of all interviewed mothers reported they have had their breastfeeding technique checked at least once. The short time of consultations was also identified as a constraint to proper counselling according to health professionals, with one saying that "mothers have not so much time, they need to walk long distances and there is a big demand at the health centre, we cannot spend much time with each patient".

Until at least 2006, an annual one-day breastfeeding training course was held, and even though none of the interviewed health professionals had attended any of them, they had a good knowledge of EBF importance and duration as they had acquired it as part of other courses. Despite this, a lack of human resources and specific training for EBF was reported by health workers as a constraint.

The solutions proposed by health workers to overcome these obstacles are training and service provision related, including improving counselling and orientation, training at least one nurse specifically in breastfeeding, promoting and introducing breastfeeding as soon as babies are born, monitoring breastfeeding during home visits, taking the opportunity of crowded days (i.e. during vaccination days) to intensify counselling and breastfeeding promotion at the health centres, to learn how to negotiate with the mother, and to be able to adapt to every situation and learn practices for better counselling.

## Antibiotics for pneumonia intervention

### Health worker level

The majority of reported obstacles (40%) to antibiotics for pneumonia (ATB-Pn) child intervention correspond to the health worker level, and among this, 23% correspond to no-adherence to guidelines ( Table 3). It was found that guidelines and protocols are either partially followed, or not followed at all by health professionals. Some of them disagreed with the current guidelines because of what they had experienced, stating that "we have seen that if you don’t prescribe an antibiotic against pharyngitis then the child will come again into the health centre with pneumonia. This is because the majority of our patients are malnourished and the weather is cold", "sometimes, I prefer to prescribe an antibiotic for preventing a pneumonia episode", and "we try to avoid complications, our patients are from long distance communities, they don’t come again to the health centre, that is why we prescribe an antibiotic, so we cut out any infection that could be present". According to interviewed health managers, even though guidelines need to be updated, those that are available are not used by doctors, stating that "they resist to act as the protocol says so they prescribe what they want", and "nurses are the only ones adhering to protocols". The proposed solutions by health managers regarding this obstacle include updating guidelines and protocols, conducting a study to prove if cotrimoxazole is still effective, and evaluating local antibiotic resistance.

**Table 3.** Classification of identified antibiotic treatment of pneumonia (ATB-Pn) related constraints and synergies according to their target level and label together with the percentage of respondents that agreed with each label.

Target level	Constraint’s label	%	Synergy’s label	%
**Health worker**	No adherence to guidelines	23	Education and promotion	4
	Health worker’s performance	11	Health worker’s performance	15
	Monitoring and follow up of patient’s treatment intake	6		
**Mother/community**	Mothers knowledge, beliefs, attitudes and practices	28	Mothers knowledge, beliefs, attitudes and practices	46
	Treatment adherence	6		
**Political/managerial**	Private shops policies and regulations for selling antibiotics	11	Continuous supply and availability of antibiotics	16
	Conflict of interests	9	Antibiotics delivered without cost	11
	No updated guidelines	3	Existence of guides and protocols	8
	Shortage of antibiotics supplies at health centres	3		

Health workers reported that some mothers forget to give the whole treatment to the child or sometimes withhold the antibiotic after 2 or 3 days when the child improves. A key factor to achieving good adherence to treatment as identified by the health workers is to properly explain to mothers how to use the antibiotic and also to speak with them in Quechua so they can understand better. Also in order to facilitate communication with mothers, health workers stressed the fact that they must consider local practices and beliefs which are not directly against the intervention, but need to be addressed for improving adherence to treatment, with one stating that "if they want to give alcohol or egg to the child we cannot prohibit this because if we are against those practices they don’t listen anymore and don’t accept the antibiotic". According to health workers and managers, communication skills need to improve in order to improve the mother’s counselling, explaining to them the importance of taking the whole treatment.

The first dose of antibiotic is usually not given at the health centre, supported by the testament of one respondent, "the antibiotic is prepared and given only to mothers that don’t understand well how to give it". According to field observations, the person in charge of delivering the antibiotic at the health centre’s pharmacy reads the prescription and re-explains to the mother how to administer the antibiotic, and an interviewed mother could recall correctly how to prepare the antibiotic, how many doses per day and for how long she must give it to her child.

According to health managers, the monitoring and follow up of patients’ treatments should be improved, with comments along the lines of, "we don’t know if patients are adhering to and following the whole treatment", and "we need to improve the monitoring and follow up of each patient’s treatment, we also need to find a place in the health centre for providing the antibiotic". Health professionals reported that even though they ask mothers to bring the child to the health centre for monitoring after 48 hours, they don’t bring them. Health workers express the need to increase the number of home visits and monitoring in the communities; for that they suggest training CHWs to follow up the fulfilment of the child’s treatment.

### Mother/community level

Antibiotics for pneumonia are provided free of charge at health centres on presentation of the prescription, and seem to be well accepted by the local population. A big concern among health managers and health workers is that mothers, instead of going to the health centre, go to a private shop or pharmacy to get antibiotics because they don’t trust the health centres. Another associated problem reported by health professionals is that pharmacies and other private shops sell antibiotics without prescriptions: "the pharmacy sells the antibiotic for a one day treatment and then the child comes to the health centre with pneumonia". Health workers and managers proposed solutions to this obstacle include regulating and restricting the sale of antibiotics in private shops and pharmacies, and educating mothers to address local beliefs and practices.

Health managers are noted to have been working on the promotion and education of mothers to improve their knowledge regarding pneumonia’s signs and symptoms. Most of the interviewed mothers recalled quite well when to seek care regarding pneumonia: "when my baby gets tired or breaths fast, I bring him to the health centre", and "cold, fast breathing and heart beats; when that happens I bring my child to the health centre" were some symptoms provided by interviewed mothers.

### Political/managerial level

Even though during the three visits to health centres, the supply and availability of antibiotics was good, health workers expressed that sometimes they have a shortage of supplies, especially in the cold season.

The local health insurance which finances the health system requests IMCI guidelines and protocols are followed for prescribing antibiotics, but to justify the cost of the child’s consultation, health professionals feel a need to prescribe. Health managers suggested that these political decisions bias the procedure, saying that, "health workers do not know what to do, because they are aware of the need of generating income for the health centre and that this is only possible through prescription", and "it is not compatible what we say and what the health insurance request we do".

Another problem reported by health professionals is that the work of some NGOs are in disagreement with IMCI guidelines, with one respondent reporting that "some NGOs are delivering antibiotics to the communities in an irrational way, not aligned with IMCI protocols, and this can cause antibiotic resistance". It was stressed by health professionals the importance to establish a collaborative action enhancing alliances and agreements to overcome this obstacle.

## IMCI intervention

### Health worker level

Most of IMCI detected constraints were at the health worker and health centre level ( Table 4). A lack of staff, high workload, and low job satisfaction were found to be obstacles affecting IMCI intervention. Health managers attributed the high workload to the fact that the population is growing, and yet the number of health workers remains the same. Health workers reported that the high number of consultations and the high load of administrative work have led to a short time per consultation, with one person responding that "on market days, time is scarce not only because of the amount of patients but also because of the filling of health insurance administrative forms which take time".

**Table 4.** Classification of identified integrated management of childhood illness (IMCI)-related constraints and synergies according to their target level and label together with the percentage of respondents that agreed with each label.

Target level	Constraint’s label	%	Synergy’s label	%
**Health worker**	Lack of staff	9	Skills and performance	12
	High work load	7	Community health workers	7
	Lack of incentives	6	Supervision	5
	Low moral of staff	4	Training	5
	Poor commitment	3		
	Lack of proper reinforcement training	2		
	High turnover of trained staff	2		
**Health centre**	Long waiting time	10	Affordability	5
	Availability	9	Availability	10
	Accessibility	7	Monitoring the drugs and vaccines supplies	2
	User satisfaction	4	User satisfaction	3
	Infrastructure	2		
**Political/managerial**	Referral system	11	Annual operational plan	2
	Collaborative action	4	Collaborative action	15
	Financing	3	Community-IMCI	2
	Institutionalization	2	Selected areas of implementation	2
	Health information system	2	Health information system	2
	Selected areas of implementation	1		
**Mother/community**	Mothers knowledge, beliefs, attitudes and practices	8	Mothers knowledge, beliefs, attitudes and practices	17
	Low economical resources	4	Education and promotion	10

The short time for consultation and the shortage of personnel not only affect the delivery of care at the health centre but also at the community level. Even though health managers report that there are already complete teams of doctors, nurses and obstetricians at the health centres, and that they have the possibility to go to long distant communities, health workers disagree, arguing that, "because of a shortage of personnel we cannot go more frequently to the communities to perform follow up activities"; "due to the high administrative work, we need to make the consultation faster, so there is no time for checking if the mother has understood what we said", and "theoretically, we should perform between 6–7 community visits per month but that is difficult to achieve with the low amount of personnel". Among mothers this obstacle was also found, with one reporting that "sometimes there are two doctors but one of them goes out to the community so only one stays at the health centre and we have to wait too long for the consultation".

A lack of staff is also affecting the supervision of activities, and health managers acknowledged a low frequency in those activities. Health professionals reported that they had been supervised for the completion of the clinical registry form but not for the clinical consultation.

The high turnover of trained staff associated with low job satisfaction caused by a lack of incentives and poor working conditions has also been reported, corroborated by responses such as "there is a frequent change of personnel, those who are trained go and new people come"; "personnel leave because of low salaries, there are no economic nor moral incentives, that is why they are not motivated", and "the administrative work as well as the community work which take a lot of time are not counted in the productivity, only the number of children that I attend in the consultation is counted". Poor commitment of the staff was also reported by health managers, which could be related to the low morale. Health professional and manager-proposed solutions include the regulation of contracted personnel working longer than 3 months to include the administrative work and travel time to communities as paid work, and to increase the number of health professionals according to population growth, especially on market days.

Lack of incentives and low morale is also present among CHWs. The role of CHWs has changed; previously they were able to implement health interventions, but nowadays they perform only preventive and promotional work. According to health professionals this has affected their image among the community, with one respondent claiming that "now CHWs have less recognition and more rejection among the community". Some mothers support this idea, stating that "there are CHWs, but they don’t know as much as they knew before and they don’t participate much. We are obliged to have our deliveries here at the health centre". However, others had a good view of the CHWs, with some saying that "when something happened to my child, I consult with the CHW and he helps me with the solution" or "I am very satisfied with the work of the CHW, he visits me and gives me advice on how to take care of my baby". As health professionals consider the work of CHWs very important, they propose increasing their training and supervision.

Facilitators to IMCI intervention reported by health professionals are IMCI training, clinical experience, speaking the Quechua language, and team work. IMCI training is being performed twice a year and is given to doctors, nurses and obstetricians, but according to health managers and professionals there is a need to pool funds to increase and update training, including two weeks of field training and neonatal-IMCI.

### Health centre level

Medical attention at health centres in this region is free for children under five years old. However, the long waiting times for consultations were reported by most of the interviewed mothers and health workers as an obstacle related to the lack of staff, high workload, and the high administrative work, as mentioned at the previous level. According to health managers, a long waiting time for the consultation could be one of the reasons for not seeking consultation at the health centre and some mothers support this fact, with one stating that, "it takes too long for the medical assistance and then when you are in, it is very fast".

Even though all visited health centres are open every day from 8 am to 8 pm and in case of emergencies 24 hours a day, some mothers reported obstacles, labelled as availability of service provision, with responses such as, "once I came to the health centre and I didn’t receive care because there were too many patients", and "last August there was a strike at the health centre, it lasted for a whole week and during that week they didn’t provide medical attention".

Accessibility has also been reported as an obstacle affecting the mothers’ ability to come to the health centre, with some claiming that, "it is difficult to come to the health centre because I need to walk and it is far away", and, "it is difficult to come to the health centre especially in an emergency at night when I need to walk through the darkness". Accessibility also affects the ability of health professionals to visit communities, especially those communities not accessible by car, and they therefore they sometimes have to walk for hours. A health professional suggested that motorcycles could help them to access those communities. Health managers are aware of the present geographical constraints, with one stating that "accessibility has been improved but still there are places where there is a need to establish health centres". Even though accessibility problems are present, mothers still come to the health centre and while some of them are pleased with the medical attention, stating that, "they give us good attention, they have a lot of patience and explain everything to us all", others are not satisfied, claiming that, "sometimes they tell me that I came only for taking advantage of the health insurance and because I want free medicines, but this disturbs me because it is not true", "there are doctors who don’t give good care or good prescriptions", and "the health personnel should work more motivated".

The availability of drugs and vaccines in all visited health centres was good. Poor infrastructure of health centres was considered by health workers as a limiting factor affecting their performance. Also the lack of well-functioning resources and equipment such as defective scales, having only one stretcher and one nebuliser, no wheelchair, and a lack of clinical registration forms, were reported by health professionals as a constraint. The lack of resources was also reported as an obstacle by the CHWs for the communities. In order to better help, especially in an emergency, they expressed the need to be supported with more resources, saying that, "we don’t have a cellular phone nor a radio to call to the health centre in case of an emergency, we need to walk to the health centre most of the times at night without having any lantern, waistcoats or rain capes". Health professionals suggest providing CHWs with cellular phones or radios to improve their communication with the health centre during emergencies.

### Political/managerial level

Under this level most obstacles point toward the referral system, particularly difficulties at the hospital level, in communication, and with the transportation system. According to health managers and health workers some hospitals reject referrals, stating that "they say they do not have space and sometimes we think they have"; "the people at the hospital are not involved and committed to the work and they don’t know how the referral system should work", and "there is an emergency and the hospital says they don’t have any bed, or we call and they don’t answer, all that makes a waste of time". Health workers also stated that there is a deficient communication system between health centres and the hospital because radios are not working well, with one claiming that, "sometimes the radio has a bad sound or no sound at all, so we can’t understand each other". Besides these obstacles, the referral system is affected by the transportation system because there are not enough ambulances or available drivers, with one stating that, "we have two drivers for the ambulance but sometimes one is on leave and the other is driving health workers to the communities, so we don’t have a driver when there is an emergency". According to health workers, the contra-referral system also has difficulties, examples being, "after hospital care, the patient comes back with a prescription directly to his home instead of coming to the health centre for a proper follow up"; "the contra-referral fails, they don’t send the patient back or the mother doesn’t bring the child back to the health centre", and "sometimes the contra-referral form is not correctly filled in". Problems have also been reported with coordinated referral, "when we set a specialist consultation date at the hospital for a patient, the mother doesn’t take the child to it, this could happen because the mother needs the authorization from the father or she needs money to pay for public transportation to the hospital and she doesn’t have it". To overcome these obstacles, health professionals and managers suggest a need to improve the administration of hospitals (including bed occupancy rates and the reception of referrals), improving management of available resources (including technical personnel), improving the referral and contra-referral system, and improving communication by phone or radio between health workers for better medical care and patient follow up.

Under collaborative action, facilitating factors such as the presence of governmental programs, NGOs, the health insurance system, local Community Associations for the Administration of Health (CLAS) (where the health centre manages its resources together with community representatives), as well as the help of CHWs were reported. A governmental program that started in 2007 has already had an impact on the monitoring of healthy children, with respondents quoted as saying, "this program gives an amount of 100 Soles to each family, but the condition is that they should bring the child to the health centre for monitoring and vaccination", and "there is an NGO that helps CHWs with teaching methodologies and they also pay for snacks and lunches".

According to some health managers, the collaborating NGOs are in line with the IMCI. Others think that the work should be of a better standard, with some reporting that, "some NGOs and governmental programs help with food donation, the problem is that mothers only give that type of food to the child, without giving anything else, then the child is not well nurtured and gets tired of eating the same thing", and "some families receive double donations while others who are also in need don’t receive any".

Other obstacles reported by health managers relate to other authorities and ministerial priorities: "the Ministry of Finance and Economics does not prioritise the same as we do", and "authorities responsible for the population have other priorities, they don’t support much health issues", being two examples provided. The lack of specific budget lines and fund identification, as well as no proper institutionalisation was identified by health managers as obstacles for the implementation of IMCI, with some stating that, "there is not much of a budget for training and follow ups", and "the IMCI health system component is too weak, and we need more support". On the other hand facilitating factors such as having an annual operational plan and the strength of community-IMCI were reported. The fact that only a few areas were selected for the IMCI implementation was reported as an obstacle, but at the same time as a facilitator, with respondents stating that, "not every health centre has IMCI, those doctors who have not been trained with IMCI, do not accept it", and "the work has been prioritized to those areas with higher risk and mortality". Regarding health information systems, health managers reported that even though a validated information system for clinical registries exists, there is always something missing or something to correct when the forms come. Health managers’ proposed solutions to overcome these obstacles included performing an updated IMCI situational analysis, to institutionalise the IMCI, to implement IMCI in all health centres, to train all health workers, to unify all nutritional supportive programs with the same cause, and to increase the commitment and involvement of the authorities.

### Mother/community level

Health workers think that health interventions are culturally accepted, but that the population acts according to its own beliefs and trusts their natural medicine as a first line treatment. Interviewed mothers have verified that, "when my child has a fever, I pass urine on him and give him fresh mate to drink and the fever goes", and "when my child has diarrhoea, I give her everything fresh and cold, like lemon-mate and Ayrampo". Other mothers go directly to the pharmacy to buy medicines, one reporting that "when my child had fever, I bought a small pink pill in the pharmacy and the fever went and since then he has not had any more". According to health workers and managers, local practices and beliefs and low economic resources are influencing the way mothers seek care, with responses such as, "because of the low education level and beliefs, they still seek for care but a bit late";, "there is some resistance when we need to hospitalise a child or administer an intravenous treatment, mothers think that we are hurting the child and they do not like to stay at the health centre because they have animals and other children to take care of", and "even though people receive economic and food support from several institutions, they don’t care about the health of their children, they sell what they get".

According to health workers, community education and promotion have good results, with reports such as "we have a mobile television with an IMCI video in Quechua that we take to the communities and show it", and "we are sensitising the communities and we are getting involved, we are starting to know each other". Even though some mothers were illiterate, they knew when to come for the next visit. Most interviewed mothers had the vaccination and IMCI card with them.

Even though health managers reported that it is still difficult to effectively teach the mother to recognize alarm symptoms, most of the interviewed mothers had a correct idea of when to come back to the health centre, stating that, "if my baby doesn’t get better from diarrhoea, or doesn’t breastfeed or cry I have to bring him again to the health centre". Most of the interviewed mothers could properly recall what health workers have recommended in terms of treatment and nutrition, but there are some foods that they cannot get because of socio-economic reasons such as, "I should give daily eggs, cheese, milk and chicken, also green vegetables and add a little oil. She usually eats potatoes and soup and sometimes I give her fruits but not much, only when I go on Sunday to the market. When we have some money, we try to buy what they recommend and give it to the child or also when we kill an animal" or "they told me to add cheese, milk, eggs and vegetables and to feed my child 6 times a day to have a good weight. I give her what we produce on our farm: potatoes, wheat, barley, cabbage, carrots and chard". According to health workers and managers’ proposed solutions, it is important to improve counselling of mothers on child nutrition and on the exchange and sale of food products in order to support their establishment in cooperatives, and to increase their income potential.

## Discussion

As far as we know this is the first study that, in order to scale up child interventions, systematically assesses constraints, synergies and possible solutions to the implementation of exclusive breastfeeding for 6 months, antibiotics for pneumonia, and IMCI child interventions in selected communities of the Cusco region, Peru. The inclusion of key informants at different levels of the health system allowed a more comprehensive and well-rounded picture of the implementation of the selected interventions and the feasibility to scale them up. The IMCI strategy includes among its components exclusive breastfeeding and antibiotics for pneumonia child interventions. The reason for assessing them independently was to increase the possibility of detecting constraints, synergies and solutions at all health system levels in order to scale them up.

### Exclusive breastfeeding for 6 months

Mothers from this region practice breastfeeding but not exclusively. Even though there are facilitators, beliefs and local practices make mothers choose a mixed feeding practice. In order to scale up EBF it is important to intensify EBF´s educational and promotional components, involving mothers, families, new generations of scholars, teenagers, health workers and authorities. Health workers are a key pillar when trying to improve this intervention. It appears that BF training for health workers is required to improve the counselling and communication skills with mothers. Also time management and organizational skills for health workers are needed in order to better allocate resources for counselling, home visits and field activities. Nankunda *et al.* showed the importance of individual peer counselling in Uganda in scaling up exclusive breastfeeding ^17^.

The mother’s socio-economical needs, together with the lack of political support, make the feasibility of practicing exclusive breastfeeding quite difficult for working mothers. Even though there are some written supportive policies, the lack of regulation and monitoring when looking at their implementation was identified as a bottleneck. In order to overcome these obstacles and scale up the intervention we recommend strengthening supportive regulations and legislative policies to improve mother’s working conditions and the monitoring and implementation of Lactarios in health centres. The strengthening of collaborative actions to improve the mother’s economy and nutrition could also help the EBF scale up. This aligns with the findings of Bhandari *et al.* ^18^ who stated the need for a legal framework with political will, strong advocacy, enabling policies, well-defined short- and long-term programme strategy, sustained financial support, clear definition of roles of multiple stakeholders and emphasis on delivery at the community level for scaling-up exclusive breastfeeding.

### Antibiotics for pneumonia

Even though there was a good availability of antibiotics for pneumonia at the visited health centres, and these were provided free of charge, we need to take into account that there might be children in the region who need antibiotics for pneumonia and who never reach a health facility. A cross-sectional study of two other regions in Peru showed that poorer households consulted less frequently for pneumonia symptoms and used less antibiotics than less poor households ^19^. According to the Indian National Health Survey 2006, only 12.5% of children received antibiotics for pneumonia when needed ^20^.

In the studied health centres, we detected room for scaling up antibiotics for pneumonia intervention, in strengthening their rational use by updating guidelines and protocols based on high-quality local studies and strengthening policies for assuring the appropriate use of antibiotics. Training health professionals by reinforcing the proper use of antibiotics and resistance risk, including improving their counselling techniques and communication skills for improving adherence to treatment is also needed. A study for improving prescribing practices in Nepal showed that peer-group discussion and a bottom-up approach of supervision and monitoring, implemented through the district health system, improved prescribing practices ^21^. Another measure is to educate mothers on how to seek appropriate care and to educate them on the importance of following a whole treatment through, taking into account their own beliefs and practices. Other measures that we propose consist of unifying and strengthening the collaboration and coordination between NGOs, governmental programs, private providers and CHWs; strengthening the monitoring and follow-up care of pneumonia patients through home visits in collaboration with CHWs; regulating the sale of antibiotics at retail sector providers; and reducing the out-of-pocket payments in the health insurance system.

### IMCI intervention

IMCI is a very complex intervention with many components, which is accepted globally as one of the key strategies to reduce child mortality ^22^. The findings of this study add to previously identified constraints of IMCI implementation in Peru as well as globally ^22, 23^. Based on the present analysis, it is possible to formulate the following recommendation in order to scale up IMCI in the Cusco region: at the political and managerial level we recommend to institutionalise IMCI and implement it in all health centres; to reform health workers’ contracting and procurement procedures; to strengthen the health information system; and to intensify the coherent implementation of collaborative actions. At the health centre and health system level our recommendation is to increase health worker staffing and improve the distribution and management of human resources; to improve working conditions and incentives to retain health workers; to provide material resources including communication technology and vehicles for facilitating community workers access to health centres; to support the work of CHWs with basic resources; and to improve the referral system, in particular the reception of patients and referrals to hospitals. At the health worker level our recommendation is to train and supervise health professionals; to train and supervise CHWs; to improve skills for mothers’ counselling; and to add a mobile support team at the health centre for days with high demand and on other days for performing community and home visits. At the mother and community level tour recommendation is to strengthen collaborative action for poverty alleviation; and to continue general education and promotion of child health.

### Study limitations

While we successfully interviewed health managers, health workers and mothers from three selected health centres, it is possible that we missed important data, especially from mothers who did not participate as we interviewed only those who came that day with their child for the consultation. There are mothers in the communities who do not consult health centres; it would be interesting to undertake interviews in the community instead of only at health centres.

The influence of external observers on the behaviour and practices of health personnel is well documented ^24^, and therefore the observed performance might differ from their usual performance. We addressed this issue by combining observational-recording with interviews, in order to obtain more precise information on the usual characteristics of child consultation and management in those health centres.

This case study focuses on current events and can provide theoretical generalizations and solutions on how these selected interventions could be scaled up but they do not permit statistical generalizations.

## Conclusions

This case study from the Cusco region in Peru demonstrates that it is feasible to scale up exclusive breastfeeding, antibiotics for pneumonia and IMCI interventions in poverty-stricken rural areas of a low-income country. The need for a coherent multi-sector approach that includes regulation, implementation and monitoring of health policies as well as education of all involved stakeholders was apparent. This study also demonstrates that global health interventions such as EBF, AB-Pn and IMCI need to undergo local adaptation. Identifying local constraints and facilitating factors in a systematic way as proposed in this study is a useful step to increase their effectiveness and reach at the local level and to identify areas for improvement in the original intervention policies. This requires feedback loops to be built-in to get the information back from the local health centres to national Ministries of Health and to the international organisations developing global guidance in the first place, such as WHO and UNICEF.
