# Supplementary material for: Constraints, synergies and avenues for scaling up breastfeeding, antibiotics for pneumonia and IMCI interventions in the Cusco region, Peru
Source: F1000Res. 2012 Dec 5;1:60. [Version 1] doi: 10.12688/f1000research.1-60.v1 (PMC3790604; doi:10.12688/f1000research.1-60.v1)
Supplement: Interview guide and checklist (Spanish) — The interview guide and checklist used to record constraints, synergies and solutions to scaling up exclusive breastfeeding, antibiotics for pneumonia and intergrated management of childhood illness (IMCI) interventions in the Cusco region, Peru. [file f1000research-1-230-s0000.tgz › Appendix_1_Interview_guide_and_check_lists.pdf]

Protocolo de Estudio para la Organización Mundial de la Salud: Complejidad de la intervención de la estrategia AIEPI con respecto a componentes individuales: como maximizar sinergias para potenciar su adecuada implementación.

INSTRUMENTO GUIA

1. Entrevistas: pool de preguntas abiertas

A) GERENTES REGIONALES DE SALUD

1. ¿Cuales son las principales **causas de muerte** en niños menores de 5 años en esta región?
2. ¿Qué **programas para la prevención** de enfermedades infantiles están funcionando actualmente en esta región?
3. ¿Que **programas para el control**-tratamiento de enfermedades infantiles están funcionando actualmente en esta región?
4. ¿Conoce la estrategia **AIEPI**? ¿Que le **parece**?
5. ¿Hay un **presupuesto** propio para AIEPI?
6. ¿Hay un **plan operativo** anual para implementar AIEPI?
7. ¿Cómo se **ejecuta** el plan operativo?
8. A nivel de profesionales de la salud, ¿se han **capacitado trabajadores** de la salud con la estrategia AIEPI en esta región?
9. ¿**A quienes**, a qué tipo de trabajadores de salud se dirige específicamente la capacitación?
10. ¿Cuántas **veces**? Recuerda cuando fue **la ultima** vez?
11. ¿Cuánto **duró** cada curso de capacitación en AIEPI?
12. ¿Cómo se han **coordinado** los cursos, nacional, regional o localmente?
13. Cuentan con **supervisores regionales** para la supervisión de personal capacitado? Y **locales**? ¿Cuantas veces supervisaron **en el último año**?
14. ¿Hubo algún **curso de actualización** desde la última capacitación en AIEPI?
15. A nivel comunitario, ¿se capacitan **promotores de salud para AIEPI comunitario**? ¿Con qué **frecuencia**? ¿Cuántos cursos **en el último año**?

15ª. ¿Qué piensa de los **promotores**?

16. ¿Cree usted que se le da la misma **importancia** a AIEPI comunitario (actividades para la comunidad) que a AIEPI clínico (capacitación profesionales de la salud)?
17. ¿Cuales son en su opinión los <u>principales obstáculos</u> para la implementación de AIEPI? ¿A nivel de **trabajadores de la salud**, a nivel de **usuarios** y a nivel **político-gerencial**?

    ¿Que se necesitaría **para mejorar** la implementación?

    ¿**A qué nivel** se podrían solucionar los problemas?

    ¿Quiénes cree usted que serían los **responsables** de implementar las soluciones?

    -Comentarios

<u>**Lactancia**</u>

18. ¿Las madres de esta región practican **lactancia materna exclusiva por 6 meses**?
19. ¿Puede estimar el **porcentaje** aproximado? ¿Por qué **motivos**?
20. ¿Hay **capacitación** especifica para el personal de salud sobre lactancia materna? Cuando fue la **última vez**?
21. ¿Puede estimar el **porcentaje** de trabajadores de la salud en esta región capacitados para promover la lactancia?
22. ¿Hay en esta región **políticas de apoyo** a la lactancia materna? **Cuales** son?
23. ¿Funciona algún tipo de **ONG, Iniciativas Nacionales o Internacionales** para promover la lactancia en la región?
24. ¿Se realiza **consejeria postnatal** para promover la lactancia?
25. ¿Y **visitas domiciliarias**?
26. ¿Hay reglamentación para las madres que **trabajan**? ¿**Cuales**?
27. ¿En los centros/puestos de salud se proporcionan **formulas**?

    A quienes? Son **gratuitas**? Hay buen **abastecimiento**?
28. ¿Cuales son en su opinión los <u>principales obstáculos</u> para la implementación de la lactancia materna exclusiva?

    Le parece que hay algo que se podría **mejorar**?

    ¿**A qué nivel** se podrían solucionar los problemas?

    ¿Quiénes serían los **responsables** de implementar las soluciones?

    -Comentarios

ATB Pneumonia

29. ¿Le parece que las madres de esta región saben reconocer **signos y síntomas** de la neumonía en niños menores de 5 años?
30. ¿Hay **pautas/guías** nacionales para la prescripción de antibióticos contra la neumonía en menores de 5 años?
31. ¿Se **cumplen**?

31ª. ¿Cuales antibióticos son los **recomendados**?

32. ¿Los antibióticos contra la neumonía, son de **venta libre** (sin receta)?
33. ¿Quienes pueden **prescribir** antibióticos contra la neumonía (médicos, enfermeras, farmaceuticos, etc.)?
34. ¿Percibe que hay algún **problema en la prescripción** de antibióticos para la neumonía?
35. ¿Los antibióticos contra la neumonía tienen **costo** para los pacientes?
    Son **subvencionados**? Si tienen costo, en general lo pueden **pagar**?
36. ¿Le parece que hay **compra y venta** de antibióticos fuera de los establecimientos de salud o farmacias, es decir en ferias o en la calle?
37. ¿Cree que la comunidad acepta y se **adhiere** al tratamiento de la neumonía? Cree que hay alguna **barrera cultural o económica**?
38. ¿Cuál le parece que son los <u>principales obstáculos</u> para la implementación de antibióticos contra la neumonía infantil?
    ¿Qué le parece que se pueda **mejorar** en el manejo de la neumonía infantil?
    ¿**A qué nivel** se podrían solucionar los problemas?
    ¿Quiénes serían los **responsables** de implementar las soluciones?
    -Comentarios

Sistemas de Salud

39. ¿Cómo es la **accesibilidad geográfica** de los establecimientos de salud de la región?
    Si la accesibilidad es limitada, ¿cómo se podría **solucionar**?
40. ¿Para los pobladores de esta región es **culturalmente aceptable** acudir a un establecimiento de salud? ¿Cree que hay **barreras culturales/economicas**?
    ¿Cómo se podrían **solucionar**? ¿**A qué nivel** se podrían solucionar?

¿Quiénes serían los **responsables** de implementar las soluciones?

41. ¿La atención en los establecimientos de salud de la región es **gratuita**?

    Y las vacunas? Medicamentos? Exámenes auxiliares?

    Si no lo es, cuanto **cuestan**? (especificar)

    Cree que un poblador promedio **puede pagarlo**?

42. ¿Cree que el centro/puesto de salud está **disponible** cuando se necesita?

    Si no, ¿cómo piensa que podría **mejorar** la disponibilidad?

    ¿**A qué nivel** se podrían solucionar los problemas?

    ¿Quiénes serían los **responsables** de implementar las soluciones?

43. ¿Están los centros/puestos de salud muy **sobrecargados** de trabajo? Si es así, ¿cómo se podría **mejorar** el problema?

44. ¿Como se coordina el **abastecimiento** de drogas, vacunas y equipos en los centros/puestos de salud?

45. ¿Esta usted satisfecho con el funcionamiento de la **logística**?

46. ¿Cómo percibe usted la **disponibilidad** de medicamentos, insumos y equipos necesarios para implementar AIEPI?

    ¿Cómo cree que se podría **mejorar la disponibilidad**?

47. ¿Hay un **sistema de información** de salud (informático o en papel) que incluya datos sobre el número de atenciones en menores de 5 años, motivos de consulta y mortalidad en menores de 5 años?

48. ¿Cómo se podría **mejorar** el sistema de información en salud?

49. ¿Hay **guías/pautas para la referencia** de pacientes? ¿Se **cumplen**?

    ¿Cómo se podría **mejorar** el sistema de referencia-contrarreferencia?

    ¿**A qué nivel** se podrían solucionar los problemas?

    ¿Quiénes serían los **responsables** de implementar las soluciones?

    -Comentarios

B) GERENTES LOCALES DE SALUD

1. ¿Cuales son las principales **causas de muerte** en niños menores de 5 años en esta comunidad?
2. ¿Han habido **cambios en la demanda de servicios**/ tasa de utilización en menores de 5 años en este centro/puesto de salud en el ultimo año? (correlacionar con los registros del establecimiento el número de consultas de menores de 5 años/día en el último año)
3. ¿Qué **programas para la prevención** de enfermedades infantiles están funcionando actualmente en este distrito?
4. ¿Que **programas para el control**-tratamiento de enfermedades infantiles están funcionando actualmente en este distrito?
5. ¿Conoce la estrategia **<u>AIEPI</u>**? ¿Que le parece?
6. ¿Hay un **presupuesto** propio para AIEPI?
7. ¿Hay un **plan operativo** anual para implementar AIEPI?
8. ¿Cómo se **ejecuta** el plan operativo?
9. A nivel de <u>profesionales de la salud</u>, ¿se han **capacitado trabajadores** de la salud con la estrategia AIEPI en este centro/puesto de salud?
10. ¿**A quienes**, qué tipo de trabajadores de salud se dirige específicamente la capacitación?
11. ¿Cuántas **veces**? Recuerda cuando fue **la ultima** vez?
12. ¿Cuánto **duró** cada curso de capacitación en AIEPI?
13. ¿Cómo se han **coordinado** los cursos, nacional, regional o localmente?
14. ¿Cuentan con **supervisores regionales** para la supervisión de personal capacitado? Y **locales**? ¿Cuantas veces supervisaron **en el último año**?
15. ¿Hubo algún **curso de actualización** desde la última capacitación en AIEPI?
16. A nivel <u>comunitario</u>, ¿se capacitan **promotores de salud para AIEPI comunitario**? ¿Con qué **frecuencia**? ¿Cuántos cursos **en el último año**?
17. ¿Que piensa sobre los **promotores**?
18. ¿Cree usted que se le da la misma **importancia** a AIEPI comunitario (actividades para la comunidad) que a AIEPI clínico (capacitación profesionales de la salud)?

19. ¿Cuales son en su opinión los principales obstáculos para la implementación de AIEPI? ¿A nivel de **trabajadores de la salud**, a nivel de **usuarios** y a nivel **político-gerencial**?
20. ¿Que se necesitaría **para mejorar** la implementación? ¿**A qué nivel** se podrían solucionar los problemas?
21. ¿Quiénes cree usted que serían los **responsables** de implementar las soluciones?
    -Comentarios

Lactancia

22. ¿Las madres de esta comunidad practican **lactancia materna exclusiva por 6 meses**?
23. ¿Puede estimar el **porcentaje** aproximado? ¿Por qué **motivos**?
24. ¿En los centros/puestos de salud se proporcionan **formulas**?
    A quienes? Son **gratuitas**? Hay buen **abastecimiento**?
25. ¿Hay **capacitación** específica para el personal de salud sobre lactancia materna?
    ¿Cuando fue la **última vez**?
    Puede estimar el **porcentaje** de trabajadores de la salud en este distrito con capacitación especial para promover la lactancia materna?
26. ¿Hay en este distrito **políticas de apoyo** a la lactancia materna? **Cuales** son?
27. ¿Funciona algún tipo de **ONG, Iniciativas Nacionales o Internacionales** para promover la lactancia en este distrito?
28. ¿Se realiza **consejeria postnatal** para promover la lactancia?
    ¿Y **visitas domiciliarias**?
29. ¿Hay reglamentación para las **madres que trabajan**? ¿**Cuales**?
30. ¿Cuales son en su opinión los principales obstáculos para la implementación de la lactancia materna exclusiva. Le parece que hay algo que se podría **mejorar**? ¿**A qué nivel** se podrían solucionar los problemas? ¿Quiénes serían los **responsables** de implementar las soluciones?
    -Comentarios

<u>ATB Pneumonia</u>

31. ¿Le parece que las madres de la comunidad saben reconocer **signos y síntomas** de la neumonía en niños menores de 5 años?
32. ¿Dónde **consultan**?
33. ¿Hay **pautas/guías** nacionales para la prescripción de antibióticos contra la neumonía en menores de 5 años? ¿Se **cumplen**?
34. ¿Los antibióticos contra la neumonía, son de **venta libre** (sin receta)? ¿Quienes pueden **prescribir** antibióticos contra la neumonía (médicos, enfermeras, farmaceuticos, etc.)?
35. ¿Percibe que hay algún **problema en la prescripción** de antibióticos para la neumonía?
36. ¿Los antibióticos contra la neumonía tienen **costo** para los pacientes, son subvencionados? Si tienen costo, en general lo pueden **pagar**?
37. ¿Le parece que hay **compra y venta** de antibióticos fuera de los establecimientos de salud?
38. ¿Cree que la comunidad acepta y se **adhiere** al tratamiento de la neumonía? Cree que hay alguna **barrera cultural**?
39. ¿Cuál le parece que son los <u>principales obstáculos</u> para la implementación de antibióticos contra la neumonía infantil?
40. ¿Qué le parece que se pueda **mejorar** en el manejo de la neumonía infantil? ¿Cómo se podría hacer las mejoras? ¿**A qué nivel** se podrían solucionar los problemas? ¿Quiénes serían los **responsables** de implementar las soluciones?
    -Comentarios

<u>Sistemas de Salud</u>

41. ¿Piensa usted que el centro/puesto de salud es **geográficamente accesible**? Si la accesibilidad es limitada, ¿cómo se podría **solucionar**?
42. ¿Piensa usted que para los pobladores de esta comunidad es **culturalmente aceptable** acudir al centro/puesto de salud? ¿Cree que hay **barreras culturales o economicas**? ¿Cómo se podrían **solucionar**? ¿**A qué nivel** se podrían solucionar? ¿Quiénes serían los **responsables** de implementar las soluciones?

43. ¿La atención en el centro/puesto de salud es **gratuita**?
Y las vacunas? Medicamentos? Exámenes auxiliares?
Si no lo es, cuanto **cuestan**? (especificar)
Cree que un poblador promedio **puede pagarlo**?
44. Si requieren **transporte** para llegar al centro/puesto de salud, ¿cree usted que el **costo** es accesible para la población tributaria de este centro/puesto?
45. ¿Cree que el centro/puesto de salud está **disponible** cuando se necesita?
Si no, ¿cómo piensa que podría **mejorar** la disponibilidad?
¿**A qué nivel** se podrían solucionar los problemas?
¿Quiénes serían los **responsables** de implementar las soluciones?
46. ¿El centro/puesto de salud está muy **sobrecargado** de trabajo?
Si es así, ¿cómo se podría **mejorar** el problema?
47. ¿Como se coordina el **abastecimiento** de drogas, vacunas y equipos en este centro/puesto?
48. ¿Esta usted satisfecho con el funcionamiento de la **logística**?
49. ¿Cómo percibe usted la **disponibilidad** de medicamentos, insumos y equipos necesarios para implementar AIEPI?
¿Cómo cree que se podría **mejorar la disponibilidad**?
50. ¿Hay un **sistema de información** de salud (informático o en papel) que incluya datos sobre el número de atenciones en menores de 5 años, motivos de consulta y mortalidad en menores de 5 años?
¿Cómo se podría **mejorar** el sistema de información en salud?
51. ¿Hay **guías y pautas para la referencia** de pacientes? ¿Se **cumplen**?
¿Cómo **funciona** el sistema de referencia y contrarreferencia?
¿**Hacia donde** se hace la referencia?
A qué **distancia** queda / cuanto **tiempo** de traslado?
¿Como se **transportan** los pacientes?
¿Tienen **ambulancia** u otro medio de transporte en el centro/puesto de salud?
¿Cómo se podría **mejorar** el sistema de referencia-contrarreferencia?
¿**A qué nivel** se podrían solucionar los problemas?
¿Quiénes serían los **responsables** de implementar las soluciones? **-Comentarios**

C) MEDICO CON FUNCION CLINICA Y GERENCIAL

1. ¿Cuanto **tiempo** hace que usted trabaja en este centro/puesto de salud?
2. ¿Cuales son las principales **causas de muerte** en niños menores de 5 años en esta comunidad?
3. ¿Han habido **cambios en la demanda de servicios**/ tasa de utilización en menores de 5 años en este centro/puesto de salud en el ultimo año? (correlacionar con los registros del establecimiento el número de consultas de menores de 5 años/día en el último año)
4. ¿Están siendo implementados actualmente **programas para la prevención** de enfermedades infantiles en este distrito? **Cuales** son?
5. ¿Están siendo implementados actualmente **programas para el control**-tratamiento de enfermedades infantiles en este distrito? **Cuales** son?
6. ¿Conoce la estrategia **<u>AIEPI</u>**? ¿Que le parece?
7. ¿Hay un **presupuesto** propio para AIEPI?
8. ¿Hay un **plan operativo** anual para implementar AIEPI?
   ¿Cómo se **ejecuta** el plan operativo?
9. A nivel de <u>profesionales de la salud</u>, ¿se han **capacitado trabajadores** de la salud con la estrategia AIEPI en este centro/puesto de salud?
   ¿**A quienes**, qué tipo de trabajadores de salud se dirige específicamente la capacitación?
   ¿Cuántas **veces**? Recuerda cuando fue **la ultima** vez?
10. ¿Cuánto **duró** cada curso de capacitación en AIEPI?
11. ¿Cómo se han **coordinado** los cursos, nacional, regional o localmente?
12. Cuentan con **supervisores regionales** para la supervisión de personal capacitado? Y **locales**? ¿Cuantas veces **en el último año**?
13. ¿Hubo algún **curso de actualización** desde la última capacitación en AIEPI?
14. A nivel <u>comunitario</u>, ¿se capacitan **promotores de salud para AIEPI comunitario**? ¿Con qué **frecuencia**? ¿Cuántos cursos **en el último año**?
15. ¿Que piensa sobre los **promotores**?
16. ¿Cree usted que se le da la misma **importancia** a AIEPI comunitario (actividades para la comunidad) que a AIEPI clínico (capacitación profesionales de la salud)?

17. ¿Cuales son en su opinión los principales obstáculos para la implementación de AIEPI? ¿A nivel de **trabajadores de la salud**, a nivel de **usuarios** y a nivel **político-gerencial**?
    ¿Que se necesitaría **para mejorar** la implementación?
    ¿**A qué nivel** se podrían solucionar los problemas?
    ¿Quiénes cree usted que serían los **responsables** de implementar las soluciones?
    -Comentarios

Lactancia

18. ¿Usted recomienda a las madres que den **lactancia materna exclusiva**?
19. ¿Por cuanto **tiempo**? **Por qué**?
20. ¿Las madres siguen su **consejo**? ¿Cuál cree que es el **motivo**?
21. ¿Cómo se podría **mejorar**?
    ¿**A qué nivel** se podrían solucionar los problemas?
    ¿Quiénes serían los **responsables** de implementar las soluciones?
22. En la consulta, ¿usted verifica la **técnica** de lactancia?
23. ¿Ante dificultades en la adherencia a la lactancia exclusiva, **insiste** en ello? **Por que**?
24. Si la madre no puede amamantar, ¿Qué es lo que usted le **recomienda**? (RN, 3m, 6m)
25. ¿Hay en la comunidad **mitos y creencias** sobre la lactancia y el calostro? En caso afirmativo, por favor de algún **ejemplo**. Usted **los aborda** en la consulta?
26. En este centro/puesto de salud se proporcionan **formulas**?
    A quienes? Son **gratuitas**? Hay buen **abastecimiento**?
27. ¿Usted ha **asistido** a un curso especifico de lactancia?
    Se hacen **cursos** de lactancia para el personal de la salud? Cuando fue **el ultimo**?
28. ¿Hay en este distrito **políticas de apoyo** a la lactancia materna? **Cuales** son?
29. ¿Funciona algún tipo de **ONG, Iniciativas Nacionales o Internacionales** para promover la lactancia en este distrito?
30. ¿Se realiza **consejeria postnatal** para promover la lactancia? ¿Y **visitas domiciliarias**?
31. ¿Hay reglamentación para las **madres que trabajan**? ¿**Cuales**?

32. ¿Cuales son en su opinión los <u>principales obstáculos</u> para la implementación de la lactancia materna exclusiva.
    ¿Le parece que hay algo que se podría **mejorar**?
    ¿**A qué nivel** se podrían solucionar los problemas?
    ¿Quiénes serían los **responsables** de implementar las soluciones?
    -Comentarios

<u>**ATB Pneumonia**</u>

33. ¿Le parece que las madres de la comunidad saben reconocer **signos y síntomas** de la neumonía?
34. ¿Es este el **primer lugar de consulta**?
35. ¿**Consultan precozmente**?
36. ¿Dan antibióticos a sus hijos **por su cuenta** antes de consultar?
37. ¿Hay **pautas/guías** nacionales para la prescripción de antibióticos contra la neumonía en menores de 5 años? ¿Se **cumplen**?
38. Los antibióticos contra la neumonía, son de **venta libre** (sin receta)?
39. ¿Quienes pueden **prescribir** antibióticos contra la neumonía (médicos, enfermeras, farmaceuticos, etc.)?
40. Percibe que hay algún **problema en la prescripción** de ATB para la neumonía?
41. Los antibióticos contra la neumonía tienen **costo** para los pacientes?
    Son **subvencionados**? Si tienen costo, en general lo pueden **pagar**?
42. ¿Le parece que hay **compra y venta** de antibióticos fuera de los establecimientos de salud?
43. ¿Están siempre **disponibles** estos medicamentos en el centro/puesto de salud?
    Si no es así, ¿cómo se podría **mejorar**?
    ¿**A qué nivel** se podrían solucionar los problemas?
    ¿Quiénes serían los **responsables** de implementar las soluciones?
44. ¿Cuando **refiere** usted a un niño con neumonía?
45. ¿Cuando decide tratar una neumonía **en el domicilio**?
    ¿Que **tratamiento** utiliza?
    ¿Hay **guías/pautas** sobre este tratamiento?

46. ¿Cómo **se lo dice** a la madre?
47. ¿Se da la **primera dosis** en el centro/puesto de salud?
48. ¿La madre sigue su **consejo**? Si no, ¿cuál le parece que es el **motivo**?
49. ¿La comunidad acepta y se **adhiere** al tratamiento de la neumonía?
    ¿Cree que hay alguna **barrera cultural**?
50. ¿La madre pide o exige por si sola el antibiótico (**demanda**)?
51. ¿En este centro/puesto **vacunan** contra el sarampión?
    Y contra el **neumococo y *H. influenzae***? A **quienes** y a que **edad**?
52. ¿Cuál le parece que son los <u>principales obstáculos</u> para la implementación de antibióticos contra la neumonía infantil?
    ¿Qué le parece que se pueda **mejorar** en el manejo de la neumonía infantil?
    ¿**A qué nivel** se podrían solucionar los problemas?
    ¿Quiénes serían los **responsables** de implementar las soluciones?
    -Comentarios

<u>**Sistemas de Salud**</u>

53. ¿Piensa usted que el centro/puesto de salud es **geográficamente accesible**?
    Si la accesibilidad es limitada, ¿cómo se podría **solucionar**?
54. ¿Piensa usted que para los pobladores de esta comunidad es **culturalmente aceptable** acudir al centro/puesto de salud? ¿Cree que hay **barreras culturales o economicas**?
    ¿Cómo se podrían **solucionar**?
    ¿**A qué nivel** se podrían solucionar?
    ¿Quiénes serían los **responsables** de implementar las soluciones?
55. ¿La atención en el centro/puesto de salud es **gratuita**?
    Y las vacunas? Medicamentos? Exámenes auxiliares?
    Si no lo es, cuanto **cuestan**? (especificar)
    Cree que un poblador promedio **puede pagarlo**?
56. Si requieren **transporte** para llegar al centro/puesto de salud, ¿cree usted que el **costo** es accesible para la población tributaria de este centro/puesto?
57. ¿Cree que el centro/puesto de salud está **disponible** cuando se necesita?
    Si no, ¿cómo piensa que podría **mejorar** la disponibilidad?

¿**A qué nivel** se podrían solucionar los problemas?

¿Quiénes serían los **responsables** de implementar las soluciones?

58. ¿El centro/puesto de salud está muy **sobrecargado** de trabajo?

    Si es así, ¿cómo se podría **mejorar** el problema?

59. ¿Como se coordina el **abastecimiento** de drogas, vacunas y equipos en este centro/puesto de salud?
60. ¿Esta usted satisfecho con el funcionamiento de la **logística**?
61. ¿Cómo percibe usted la **disponibilidad** de medicamentos, insumos y equipos necesarios para implementar AIEPI?

    ¿Cómo cree que se podría **mejorar la disponibilidad**?

62. ¿Hay un **sistema de información** de salud (informático o en papel) que incluya datos sobre el número de atenciones en menores de 5 años, motivos de consulta y mortalidad en menores de 5 años?

    ¿Cómo se podría **mejorar** el sistema de información en salud?

63. ¿Hay **guías y pautas para la referencia** de pacientes? ¿Se **cumplen**?
64. ¿Cómo **funciona** el sistema de referencia y contrarreferencia?

    ¿**Hacia donde** se hace la referencia?

    A qué **distancia** queda / cuanto **tiempo** de traslado?

    ¿Como se **transportan** los pacientes?

    ¿Tienen **ambulancia** u otro medio de transporte en el centro/puesto de salud?

    ¿Cómo se podría **mejorar** el sistema de referencia-contrarreferencia?

    ¿**A qué nivel** se podrían solucionar los problemas?

    ¿Quiénes serían los **responsables** de implementar las soluciones?

65. ¿Cree que hacen falta **cursos de actualización** en manejo de enfermedades y prevención?

    -Comentarios

D) PROFESIONAL DE LA SALUD CON FUNCION CLINICA

1. ¿Cuanto **tiempo** hace que usted trabaja en este centro/puesto de salud?
2. ¿Cuales son las principales **causas de muerte** en niños menores de 5 años en esta comunidad?
3. ¿Han habido **cambios en la demanda de servicios**/ tasa de utilización en menores de 5 años en este centro/puesto de salud en el ultimo año? (correlacionar con los registros del establecimiento el número de consultas de menores de 5 años/día en el último año)
4. ¿Están siendo implementados actualmente **programas para la prevención** de enfermedades infantiles en este distrito? **Cuales** son?
5. ¿Están siendo implementados actualmente **programas para el control**-tratamiento de enfermedades infantiles en este distrito? **Cuales** son?
6. ¿Conoce la estrategia **AIEPI**?
7. ¿Usted fue **capacitado** en cursos de AIEPI?
   ¿**Cuándo**? Cuantas **veces**? ¿Cuánto **duró** el curso?
8. ¿Tomo algún **curso de actualización** desde su primera capacitación en AIEPI?
9. ¿Fue alguna vez **supervisado** en sus actividades luego de la capacitación?
   ¿Cuándo fue la **última vez**?
10. ¿Que le **parece** (que sirve)? ¿La **utiliza**? ¿**Por qué**?
11. ¿Que piensa de los **promotores** de salud (agentes comunitarios)?
12. ¿Cuales son en su opinión los principales obstáculos para la implementación de AIEPI?
    ¿A nivel de **trabajadores de la salud**, a nivel de **usuarios** y a nivel **político-gerencial**?
    ¿Que se necesitaría **para mejorar** la implementación?
    ¿**A qué nivel** se podrían solucionar los problemas?
    ¿Quiénes cree usted que serían los **responsables** de implementar las soluciones?
    -Comentarios

<u>Lactancia</u>

13. ¿Usted recomienda a las madres que den **lactancia materna exclusiva**?
14. ¿Por cuanto **tiempo**? **Por qué**?
15. ¿Las madres siguen su **consejo**? ¿Cuál cree que es el **motivo**?
    ¿Cómo se podría **mejorar**?
    ¿**A qué nivel** se podrían solucionar los problemas?
    ¿Quiénes serían los **responsables** de implementar las soluciones?
16. En la consulta, ¿usted verifica la **técnica** de lactancia?
17. ¿Ante dificultades en la adherencia a la lactancia exclusiva, **insiste** en ello? **Por que**?
18. Si la madre no puede amamantar, ¿Qué es lo que usted le **recomienda**? (RN, 3m, 6m)
19. ¿Hay en la comunidad **mitos y creencias** sobre la lactancia incluyendo el calostro?
    En caso afirmativo, por favor de algún **ejemplo**. Usted **los aborda** en la consulta?
20. En este centro/puesto de salud se proporcionan **formulas**?
    ¿**A quienes**? ¿Son **gratuitas**? ¿Hay buen **abastecimiento**?
21. ¿Usted ha **asistido** a un curso específico de lactancia?
    ¿Se hacen **cursos** de lactancia para el personal de la salud?
    ¿Cuando fue **el último** curso?
22. ¿Hay en este distrito **políticas de apoyo** a la lactancia materna? ¿**Cuales** son?
23. ¿Funciona algún tipo de **ONG, Iniciativas Nacionales o Internacionales** para promover la lactancia en este distrito?
24. ¿Se realiza **consejería postnatal** para promover la lactancia?
    ¿Y **visitas domiciliarias**?
25. ¿Hay reglamentación para las **madres que trabajan**? ¿**Cuales**?
26. ¿Cuales son en su opinión los <u>principales obstáculos</u> para la implementación de la lactancia materna exclusiva.
    Le parece que hay algo que se podría **mejorar**?
    ¿**A qué nivel** se podrían solucionar los problemas?
    ¿Quiénes serían los **responsables** de implementar las soluciones?
    -Comentarios

ATB Pneumonia

27. ¿Le parece que las madres de la comunidad saben reconocer **signos y síntomas** de la neumonía?
28. ¿Es este el **primer lugar de consulta**?
29. ¿**Consultan precozmente**?
30. ¿Dan antibióticos a sus hijos **por su cuenta** antes de consultar?
31. ¿Hay **pautas/guías** nacionales para la prescripción de antibióticos contra la neumonía en menores de 5 años? ¿Se **cumplen**?
32. Los antibióticos contra la neumonía, son de **venta libre** (sin receta)?
33. ¿Quienes pueden **prescribir** antibióticos contra la neumonía (médicos, enfermeras, farmaceuticos, etc.)?
34. ¿Percibe que hay algún **problema en la prescripción** de antibióticos para la neumonía?
35. ¿Los antibióticos contra la neumonía tienen **costo** para los pacientes?
    ¿Son **subvencionados**? Si tienen costo, en general lo pueden **pagar**?
36. ¿Le parece que hay **compra y venta** de antibióticos fuera de los establecimientos de salud?
37. ¿Están siempre **disponibles** estos medicamentos en el centro/puesto de salud?
    Si no es así, ¿cómo se podría **mejorar**?
    ¿**A qué nivel** se podrían solucionar los problemas?
    ¿Quiénes serían los **responsables** de implementar las soluciones?
38. ¿Cuando **refiere** usted a un niño con neumonía?
39. ¿Cuando decide tratar una neumonía **en el domicilio**?
    ¿Que **tratamiento** utiliza?
    ¿Hay **guías/pautas** sobre este tratamiento?
40. ¿Cómo **se lo dice** a la madre?
41. ¿Se da la **primera dosis** en el centro/puesto de salud?
42. ¿La madre sigue su **consejo**? Si no, ¿Cuál le parece que es el **motivo**?
43. ¿La comunidad acepta y se **adhiere** al tratamiento de la neumonía? Cree que hay alguna **barrera cultural**?
44. ¿La madre pide o exige por si sola el antibiótico (**demanda**)?

45. Que pasa si no se prescribe antibiótico por ser probablemente una infección viral, queda la madre desconforme?
46. ¿En este centro/puesto **vacunan** contra el sarampión?
    Y contra el **neumococo y *H. influenzae***? A **quienes** y a que **edad**?
47. ¿Cuál le parece que son los <u>principales obstáculos</u> para la implementación de antibióticos contra la neumonía infantil?
    ¿Qué le parece que se pueda **mejorar** en el manejo de la neumonía infantil?
    ¿Cómo se podría hacer las mejoras?
    ¿**A qué nivel** se podrían solucionar los problemas?
    ¿Quiénes serían los **responsables** de implementar las soluciones?
    -Comentarios

<u>Sistemas de Salud</u>

48. ¿Piensa usted que el centro/puesto de salud es **geográficamente accesible** para los pobladores?
    Si la accesibilidad es limitada, ¿cómo se podría **mejorar**?
49. ¿Piensa usted que para los pobladores de esta comunidad es **culturalmente aceptable** acudir al centro/puesto de salud? ¿Percibe **barreras culturales**? Se le ocurre algún **ejemplo**?
    ¿Cómo se podrían **solucionar**?
    ¿**A qué nivel** se podrían solucionar?
    ¿Quiénes serían los **responsables** de implementar las soluciones?
50. ¿La atención en el centro/puesto de salud es **gratuita**?
    Y las vacunas? Medicamentos? Exámenes auxiliares?
    Si no lo es, cuanto **cuestan**? (especificar)
    Cree que un poblador promedio **puede pagarlo**?
51. Si requieren **transporte** para llegar al centro/puesto de salud, ¿cree usted que el **costo** es accesible para la población tributaria de este centro/puesto?
52. ¿Cree que el centro/puesto de salud está **disponible** cuando se necesita?
    Si no, ¿cómo piensa que podría **mejorar** la disponibilidad?
    ¿**A qué nivel** se podrían solucionar los problemas?

¿Quiénes serían los **responsables** de implementar las soluciones?

53. ¿El centro/puesto de salud está muy **sobrecargado** de trabajo en las consultas para menores de 5 años?

    Si es así, ¿cómo se podría **mejorar** el problema?

    ¿**A qué nivel** se podrían solucionar los problemas?

    ¿Quiénes serían los **responsables** de implementar las soluciones?

54. ¿Siente que puede **desempeñar** óptimamente su trabajo?

    ¿Qué es lo que mas **dificulta** su trabajo?

    ¿Qué es lo que mas **facilita** su trabajo?

    ¿En que se podría **mejorar**?

    ¿**A qué nivel** se podrían solucionar los problemas?

    ¿Quiénes serían los **responsables** de implementar las soluciones?

55. ¿Cómo percibe usted la **disponibilidad** de medicamentos, insumos y equipos necesarios para implementar AIEPI?

    ¿Cómo cree que se podría **mejorar la disponibilidad**?

56. ¿Hay un **sistema de información** de salud (informático o en papel) que incluya datos sobre el número de atenciones en menores de 5 años, motivos de consulta y mortalidad en menores de 5 años?

    ¿Cómo se podría **mejorar** el sistema de información en salud?

57. ¿Hay **guías y pautas para la referencia** de pacientes? ¿Se **cumplen**?

58. ¿Cómo **funciona** el sistema de referencia y contrarreferencia?

    ¿**Hacia donde** se hace la referencia?

    A qué **distancia** queda / cuanto **tiempo** de traslado?

    ¿Como se **transportan** los pacientes?

    ¿Tienen **ambulancia** u otro medio de transporte en el centro/puesto de salud?

    ¿Cómo se podría **mejorar** el sistema de referencia-contrarreferencia?

    ¿**A qué nivel** se podrían solucionar los problemas?

    ¿Quiénes serían los **responsables** de implementar las soluciones?

59. ¿Cree que hacen falta **cursos de actualización** en manejo de enfermedades y prevención?

    -Comentarios

E) MADRES

Características de las madres entrevistadas:

-Idioma de la entrevista: ______________

-Edad de la madre: ______________

-Cantidad de hijos: ______________

-Puede leer?: ______________

-Ultimo año (nivel) de educación formal: ______________

-Profesión / oficio / trabajo: ______________

-¿Vive con otro adulto? ______________

-Motivo de consulta: ______________

-Edad del hijo por el cual consulta: ______________

- Parto en institución o en domicilio: ______________

1. ¿Qué **te dijo el medico** que tiene el niño?
2. ¿Que **cuidados** te indico?
3. ¿Le tienes que dar al niño algún **tratamiento/medicina**? **Cual**?
4. ¿Cómo tienes que **preparar el medicamento**?
5. ¿Por **cuantos días** tienes que darlo?
6. ¿Cuántas **veces al día**?
7. ¿**Por qué** tienes que darle este medicamento?
8. ¿Te dijo el medico si tienes que **controlar**/observar algo en tu niño (signos de peligro)?
9. ¿Frente a que síntomas/signos **volverías** a consultar a la posta?
10. ¿A que **distancia** vives del centro/puesto de salud?
11. ¿**Cómo vienes** al centro/puesto de salud: a pie, en carro, ómnibus, a lomo de bestia, etc.?
12. ¿Cuanto **tiempo** necesitas para llegar desde tu casa?
13. ¿Que **comió ayer** tu hijo?
14. ¿Que **come en general** tu hijo?
15. ¿Que te **aconsejó el medico** que le dieras de comer?
16. ¿Le diste la **primera leche** cuando nació? Si no, **por que**? **Que le diste** al nacer?

17. ¿Le das/ le diste **pecho**? Por cuanto **tiempo** le diste solo pecho, sin agua, mate ni ningún otro alimento? **Por qué**?
18. ¿Cuando le empezaste a dar **otros alimentos** (papillas/sopas) a tu hijo? ¿Por **cuál** empezaste? ¿**Que** le dabas?
19. ¿Te resulta/resultó **fácil darle pecho**?
20. ¿Tienes/tuviste alguna **dificultad**?
21. Si **trabaja**, ¿cómo haces/hacías para darle pecho cuando vas/ibas a trabajar?
22. ¿Te sientes/sentías **libre para darle pecho** donde querías a tu hijo?
23. ¿**Hablo el medico** contigo sobre darle pecho?
24. ¿Has recibido **visitas domiciliarias** de consejo? Si has recibido, que te **aconsejaron**?
25. ¿Te **chequean o alguna vez te chequearon** como das el pecho cuando vas a la posta?
26. ¿Conoces a alguna **madre que no haya podido dar pecho**? ¿**Por qué**?
27. Que tú recuerdes, ¿se hicieron **charlas comunitarias** sobre dar el pecho? ¿**Participaste**? ¿Qué te **pareció**?
28. ¿Tienes **apoyo** de parte de tu pareja, familia, vecinos, amigos para dar pecho y alimentar a su bebe?
29. ¿**Controlan el peso** de tu bebe en el centro/puesto de salud?
30. ¿Tiene tu hijo **la tarjeta de vacunación/control**? Podrías **mostrármelo**?
31. ¿Que haces cuando tu hijo tiene **fiebre**? Como manejas el problema **en casa**? ¿**Que le das**?
32. ¿Que haces cuando tu hijo **vomita**? Como manejas el problema **en casa**? ¿**Que le das**?
33. ¿Que haces cuando tiene **diarrea**? Como manejas el problema **en casa**? ¿**Que le das**?
34. ¿Que haces cuando tiene **tos**? Como manejas el problema **en casa**? ¿**Que le das**?
35. ¿Alguna vez tuvo **dificultad para respirar**? Como manejas el problema **en casa**? ¿**Que le das**?
36. ¿**Consultas con alguien**: vecina, pariente, curandero, promotor, farmacia, botiquín comunal etc.? Donde vas **primero**?
37. ¿Cuando decides **consultar en el centro/puesto de salud**?

38. ¿Te resulta **fácil llegar** a consultar al centro/puesto de salud?
39. ¿Te paso alguna vez que viniste al centro/puesto de salud y estaba **cerrado**?
40. ¿Te paso alguna vez que viniste al centro/puesto de salud y **no te pudieron atender**?
41. ¿Te resulta **fácil hacer** lo que te recomienda el medico?

    ¿Te ha dado buen **resultado**?
42. ¿Estas **contenta con la atención** en este centro/puesto de salud?
43. ¿Que te parece que habría que **mejorar en el centro/puesto de salud** para que la atención de tu hijo sea mejor?

    Que te parece que tienen que **mejorar los doctores**?
44. ¿Que piensas de los **promotores**? Te **ayudan** a resolver los problemas de tu hijo?
45. ¿Se hacen seminarios o **actividades educativas de salud en la comunidad**?

2. Observation-recording guide

2.1. In the pediatric consultation

-Check for general danger signs (convulsions; can not drink; vomit everything; lethargic or unconscious)

-Check the presence of cough or difficult breathing.

If yes: -count the frequency (breaths per minute).

-Look for chest indrawing

-Check the presence of diarrhea,

If yes: -check for blood in the stool

-check signs of dehidratation

-Check the presence of fever

If yes: - check the temperature

- signs of meningitis

-signs of measles (generalized rash, cough, runny nose, red eyes, mouth ulcers)

-Check for ear problem

-Weigh the child and check his weight against a recommended growth chart

-Check palmar pallor

-Check oedema of both feet

-Check the child's vaccination status

-If the child is under-2 years check breastfeeding, complementary foods and feeding practices during this episode of illness

-Need and proceed properly with urgent referral

-Need and proceed with the proper prescription of oral treatment

-Check for vaccination card or history and prescribe when necessary

-Communication skills for care and treatment at home: oral treatment: preparation, amount, how many times per day, how many days

-Check caretaker's comprehension on how to administer the oral treatment

-First dose given at the facility

-Explain when to return (danger sings)

2.2. Características generales del centro/puesto de salud:

1. ¿Qué área cubre el centro/puesto de salud (km radio y numero de habitantes)?

2. Días y horarios de atención al público pediátrico.

3. ¿Cuanta gente atienden por día?

4. ¿Hay mucha gente en la consulta (sala de espera)?

5. ¿Tienen que pagar por la atención? ¿Y por los medicamentos?

6. ¿Qué profesionales de la salud hay, profesión/cantidad?

7. ¿Hay días específicos para vacunación o se puede hacer todos los días?

8. ¿Los servicios de prevención (control del niño sano) y curación para pediatría están integrados o son independientes uno de otro?

9. ¿A que distancia esta el próximo centro/puesto de salud del mismo nivel? Y el próximo de mayor nivel?

2.3. LISTA DE DROGAS, VACUNAS Y EQUIPAMIENTOS PARA CHEQUEAR EN EL CENTRO/PUESTO DE SALUD

**Rubro**	**Comentarios** (vencimiento, cantidad)	**Tiene (√)**<br>**No tiene (-)**
**Drogas orales**		
Amoxicilina o co-trimoxazol.		
Cloroquina y Primaquina	Solo en región de malaria	
Paracetamol		
Vitamina A		
Hierro		
Mebendazol o Albendazol		
Sales de rehidratación oral		
**Drogas parenterales**		
PG Sódica.		
P. Procaínica.		
P. Benzatínica		
Cloranfenicol		
Quinina	Solo en region de malaria	
**Vacunas**		
BCG		
Sarampión		
Polio		
DPT		
TT		
H. influenzae		
Hepatitis B		
Fiebre amarilla	Sólo en zonas endémicas	
**Equipamiento**		
Balanza de bebés funcional		
Balanza de adultos funcional		
Reloj o cronómetro		
Termometro		
Bolsitas salvadoras		
Oxigeno		
Fuente de agua limpia		
Carnés de vacunación		
Tarjetas madres para el hogar		
Cuadro procedimientos AIEPI		
Transporte para referencia		
Refrigeradora o cadena de frio		
Esterilizador		
Estufas		
Jeringas/agujas descartables		
Cucharas/vasos para SRO		
