# Supplementary material for: Constraints, synergies and avenues for scaling up breastfeeding, antibiotics for pneumonia and IMCI interventions in the Cusco region, Peru
Source: F1000Res. 2012 Dec 5;1:60. [Version 1] doi: 10.12688/f1000research.1-60.v1 (PMC3790604; doi:10.12688/f1000research.1-60.v1)
Supplement: Peer review history

# Peer review: Referee response for version 1

**Published:** 2013-01-08

**Authors:** M. Rashad Massoud

**Affiliations:** 1 Department of Public Health, University Research Co LLC, Bethesda, USA

I have read this submission. I believe that I have an appropriate level of expertise to confirm that it is of an acceptable scientific standard.

# Peer review: Referee response for version 1

**Published:** 2012-12-17

**Authors:** Gill Walt

**Affiliations:** 1 Global Health and Development Department, London School of Hygiene and Tropical Medicine, London, UK

This paper provides no new insights to the issues it addresses, but it does discuss the difficulties of implementing specific child policies in particular settings in Cusco, Peru, which will have resonance for other parts of the world. There is a useful discussion on recommended actions at the various levels of the health service.

I have read this submission. I believe that I have an appropriate level of expertise to confirm that it is of an acceptable scientific standard.

# Peer review: Referee response for version 1

**Published:** 2012-12-13

**Authors:** Elizabeth Molyneux

**Affiliations:** 1 Department of Paediatrics, University of Malawi, College of Medicine, Blantyre 3, Malawi

The authors of this paper have looked at the difficulties in scaling up breast feeding and pneumonia and the IMCI interventions in a region of Peru.

They are to be commended for their ’360 degree look’ at the problems and in trying to find solutions from the workers and carers within the system. The answers are not new but need repeating; many problems could be solved if the recommendations from within the service are listened to and carried out. Other health services could ask similar questions of their health service providers and users and would probably find similar suggestions to act upon.

I have read this submission. I believe that I have an appropriate level of expertise to confirm that it is of an acceptable scientific standard.
